# C-peptide is independent associated with diabetic peripheral neuropathy: a community-based study

**DOI:** 10.1186/s13098-017-0208-2

**Published:** 2017-02-13

**Authors:** Xiaona Qiao, Hangping Zheng, Shuo Zhang, Siying Liu, Qian Xiong, Fei Mao, Zhaoyun Zhang, Jie Wen, Hongying Ye, Yiming Li, Bin Lu

**Affiliations:** 1 Department of Endocrinology and Metabolism, Huashan Hospital, Fudan University, 12 Middle Urumqi Road, Shanghai, 200040 China; 2 Departments of Endocrinology and Metabolism, Huashan Hospital Jing’an Branch, Fudan University, Shanghai, 200040 China

**Keywords:** Diabetic peripheral neuropathy, C-peptide, Beta-cell function, Community-based

## Abstract

**Background:**

Because the relationship between C-peptide and diabetic peripheral neuropathy (DPN) is controversial, the aim of our study was to evaluate the relationship between C-peptide and DPN in community-based Chinese patients with type 2 diabetes.

**Methods:**

In total, 220 consecutive type 2 diabetic patients treated by our regional medical consortium were enrolled. DPN was assessed by clinical symptoms, signs, and electromyography.

**Results:**

Fasting C-peptide, 2-h postprandial C-peptide and ΔC-peptide (i.e., 2-h postprandial C-peptide minus the fasting C-peptide) serum concentrations in the non-DPN group were significantly higher than those in the clinical DPN group (all *P* ≤ 0.040) and the confirmed DPN group (all *P* < 0.002). The three C-peptide parameters were independently associated with DPN (all *P* < 0.05) after adjusting for age, sex, diabetes duration, smoking status, systolic pressure, body mass index, angiotensin-converting enzyme inhibitors/angiotensin receptor blocker use, fasting plasma glucose, HbA1c, triglyceride and estimated glomerular filtration rate. Compared with the ΔC-peptide quartile 1 (reference), patients in quartile 3 (odds ratio [OR], 0.110; 95% confidence interval [CI] 0.026–0.466; *P* = 0.003) and quartile 4 (OR, 0.012; 95% CI 0.026–0.559; *P* = 0.007) had a lower risk of DPN after adjusting for the confounders.

**Conclusions:**

C-peptide was negatively associated with DPN in community-based Chinese type 2 diabetic patients in China.

## Background

Diabetic peripheral neuropathy (DPN) is a well-known microvascular complication of type 2 diabetes mellitus, which leads to further infections and increases the risk of foot ulcers, non-traumatic amputations and mortality [1, 2]. Although hyperglycemia plays an important role in the development of DPN, intensive glucose control does not eliminate the risk of developing DPN in patients with type 2 diabetes, suggesting that other factors may be involved in DPN development [3].

C-peptide levels in the peripheral blood are widely accepted as the most appropriate evaluation of insulin secretion, and are not eliminated in the first-pass metabolism through the liver [4, 5]. Previously considered to be an inactive by-product of insulin synthesis, C-peptide is a hormonally active peptide [5, 6]. In type 1 diabetes, an Italian study with a large clinical cohort demonstrated a significant association between C-peptide and microvascular complications, including neuropathy [7]. Several studies in animal models of diabetes and in patients with type 1 diabetes have demonstrated beneficial effects of C-peptide replacement on both peripheral and autonomic nerve function in diabetes [8–10].

In contrast to the data collected in patients with type 1 diabetes, studies on the relationship between C-peptide and in type 2 diabetes mellitus-related complications were conflicting. Whereas some studies showed that residual insulin secretion, evaluated by serum C-peptide concentrations, has a protective effect on diabetic neuropathy [11–13], others either did not find such an effect [14], or concluded a contrary relationship [15]. Moreover, none of the studies focused on a community-based population [13]. Therefore, the aim of this study was to evaluate the relationship between C-peptide levels and DPN, independent of glycemic control and other risk factors in community-based Chinese type 2 diabetic patients.

## Methods

### Subjects

In total, 220 consecutive type 2 diabetic patients receiving care from the Jing’an regional medical consortium with completed demographic information and neurological examination were enrolled in this study. World Health Organization diagnostic criteria were used for type 2 diabetes diagnoses [16]. Patients with acute complications of diabetes, renal dysfunction (estimated glomerular filtration rate [eGFR] <60 mL/min/1.73 m^2^), acute cerebral infarction, vitamin B12 deficiency, alcohol abuse and asymmetrical neuropathy of the trunk or proximal lower limbs and chronic infection were excluded from this study. All the patients signed the consent form for allowing their information to be used for research. The study was approved by the Ethics committee of Huashan Hospital, Fudan University and all patients provided informed consent.

### Anthropometric measurements

Body weight was assessed with the patients wearing light clothing and no footwear before breakfast; all heights measurements were taken using the same wall-mounted stadiometer. Body mass index (BMI) was calculated as body weight (in kg) divided by the square of the height (in m). Diastolic blood pressure (DBP) and systolic blood pressure (SBP) were measured three times with 1-min intervals after 10 min of using a standard mercury sphygmomanometer and then averaged.

### Laboratory measurements

Glucose and C-peptide concentrations were measured at baseline and 2 h following oral administration of a 100-g steamed bread meal test (equal to 75 g of glucose). ΔC-peptide levels were calculated as the 2-h postprandial serum C-peptide level minus the fasting C-peptide level. Although not a standard test in diabetes research or care, the 100 g steamed bread meal test was selected because it avoids severe glucose fluctuation in patients with type 2 diabetes mellitus [17]. Fasting blood samples were collected in a sodium fluoride anticoagulant tube, an EDTA anticoagulant tube and a coagulating tube. Glucose, glycosylated hemoglobin A1c (HbA1c) and biochemical indicators including serum creatinine (Cr), and lipid profiles including total cholesterol (TC), triglyceride (TG), high-density lipoprotein-cholesterol (HDL-C) and low-density lipoprotein-cholesterol (LDL-C) were measured. 2-h postprandial blood samples were also collected for glucose and C-peptide measurements. Fasting plasma glucose (FPG) and 2-h postprandial plasma glucose (2 h-PG) concentration was estimated using the hexokinase method (Wako pure 2756-01, Wako Pure Chemical Industries, Osaka, Japan) and an automatic analyzer (Hitachi 7600-120, Hitachi High-Technologies, Schaumburg, America). Whole blood was used to measure HbA1c by high-pressure liquid chromatography using an analyzer (HLC-723G7, Tosoh Corporation, Tokyo, Japan) and the matched calibrators and controls in a sampling volume of 200 μL. For measurements of biochemical indicators, blood was centrifuged at 3000 r/min for 10 min within 30 min of collection (5810R, Eppendorf, Hamburg, Germany) and the serum was collected for subsequent testing. Serum TC (GPO-DAOS method, Wako Pure Chemical Industries), TG (DAOS method, Wako Pure Chemical Industries), HDL-C (Direct method, Sekisui Medical Technology, Shanghai, China), and LDL-C (Direct method, Sekisui Medical Technology, Osaka, Japan) of all the patients were estimated on the analyzer (Hitachi 7600-120). Glycated albumin (GA) was measured by an enzymatic assay (Lucica GA-L, Asahi Kasei Corporation, Tokyo, Japan) on the automatic analyzer (Hitachi 7600-120) using a sample volume of 10 μL. C-peptide was measured by chemiluminescence (Siemens Healthcare Diagnostics, Malvern, USA) on an ADVIA Centaur XP automatic analyzer (Siemens Healthcare Diagnostics) using a sample volume of 200 μL. The glomerular filtration rate (GFR) was estimated using the modification of diet in renal disease (MDRD) equation recalibrated for Chinese people [18].

### Examination of neurological symptoms and signs

Neurological symptoms and signs based on the neuropathy symptom score (NSS) and the neuropathy disability scores (NDS) were evaluated [1], Neurological symptoms included burning, numbness, tingling, fatigue, cramping or aching, and neurological signs included vibration sense, pain, temperature sensation and ankle reflex. The symptoms and signs abnormalities assessed for DPN were in a glove/stoking distribution.

### Nerve conduction velocity tests

An electromyography (EMG) machine (Keypoint^®^4; Tonsbakken 16-18, DK-2740 Skovlunde, Denmark) was utilized to assess sensory and motor nerve conduction velocity (NCV) by the same neurologist. NCV studies of bilateral median, ulnar, tibial, common peroneal and superficial peroneal on each subject were conducted. Subjects stayed calm and relaxed, and the local skin temperatures were kept at 32–33 °C throughout the examination. A decrease in NCV was set according to the NCV reference value for the Chinese population [19].

### Diagnosis of DPN

DPN was diagnosed according to the modified Toronto Expert Consensus [3] as follows: (1) non-DPN, all neurological symptoms/signs and NCV were normal; (2) clinical DPN, at least two abnormal results among neurological symptoms/signs, or ankle reflex in accordance with a distal symmetrical polyneuropathy and normal NCV; (3) confirmed DPN, at least one abnormal nerve parameter (of NCV, amplitude, latency, and F-wave) in two or more nerves among the median, peroneal, and sural nerves, regardless of neurological signs and symptoms.

### Statistical analysis

Data are presented as the mean ± standard deviation or percent of individuals. Variance homogeneity was assessed by the Levene test. Differences in C-peptide within the three groups were assessed by one-way ANOVA. The least significant difference and Dunnett tests were used to compare differences between groups with continuous variables, and a Chi square test was used to assess differences between categorical variables. Spearman’s correlation analysis was used to examine the correlation of serum C-peptide concentrations with clinical variables. Multiple logistic regression analysis was performed to evaluate the association of DPN and C-peptide quartiles after adjusting for other clinical and biochemical variables. Analysis was performed using SPSS 17.0 (IBM Corporation, Somers, NY). A *P* value of <0.05 was considered to be statistically significant.

## Results

The clinical characteristics of the patients in all three groups are summarized in Table 1. Compared to patients in the non-DPN group, those in the confirmed DPN groups had higher SBP (*P* = 0.036), FPG (*P* = 0.033), 2 h-PG (*P* = 0.012) and HbA1c (*P* = 0.007) as well as lower HDL-C (*P* = 0.018), NCV and nerve conduction amplitude (NCA) of median, titial and sural (all *P* < 0.05). However, no significant differences in age, sex, diabetes duration, smoking status, BMI, DBP, TC, TG, LDL-C, GA, Cr and eGFR were found among the three groups. The use of anti-diabetic drugs (e.g., metformin, sulfonylureas and insulin) and anti-hypertension drugs [angiotensin-converting enzyme inhibitors (ACEI) or angiotensin receptor blocker (ARB)] were also not significantly different among the three groups.

**Table 1:** Characteristic of subjects

	Non-DPN (128)	*P*_1V3_	Clinically-DPN (35)	*P*_1V2_	Comfirmed DPN (57)	*P*_2V3_
Age (year)	62.3 ± 8.4	0.449	63.3 ± 10.7	0.527	64.5 ± 8.8	0.726
Sex (male %)	41%	0.568	58.40%	0.634	46%	0.62
Diabetes duration (year)	8.5 ± 7.2	0.084	9.7 ± 7.7	0.435	12.6 ± 6.9	0.228
Smoking	12 (9.4%)	0.736	4 (11.4%)	0.543	6 (10.5) %	0.612
BMI (kg/cm^2^)	25.1 ± 3.8	0.393	25.8 ± 4.7	0.421	26.9 ± 1.3	0.167
SBP (mmHg)	133 ± 16	0.036	135 ± 16	0.501	141 ± 13	0.056
DBP (mmHg)	78 ± 8	0.239	77 ± 11	0.431	83 ± 9	0.068
FPG (mmol/L)	8.40 ± 2.43	0.033	8.89 ± 2.33	0.523	9.37 ± 2.60	0.465
2 h-PG (mmol/L)	15.79 ± 4.81	0.012	16.47 ± 4.63	0.607	18.14 ± 4.81	0.263
TC (mmol/L)	5.30 ± 1.02	0.356	5.19 ± 1.06	0.737	5.10 ± 1.26	0.823
TG (mmol/L)	2.11 ± 1.17	0.742	2.38 ± 0.82	0.448	2.05 ± 1.12	0.383
HDL-C (mmol/L)	1.33 ± 0.45	0.018	1.18 ± 0.18	0.217	1.14 ± 0.22	0.788
LDL-C (mmol/L)	2.96 ± 0.77	0.906	3.02 ± 0.83	0.804	2.98 ± 1.09	0.878
HBA1c (%)	7.4 ± 1.4	0.007	7.6 ± 1.4	0.929	8.5 ± 1.9	0.229
GA (%)	18.7 ± 3.7	0.091	20.4 ± 5.2	0.417	22.8 ± 6.1	0.086
Cr (μmol/L)	61.54 ± 15.35	0.276	63.94 ± 17.11	0.63	67.6 ± 17.30	0.278
eGFR (mL/min/1.73 m^2^)	104.35 ± 31.32	0.857	106.34 ± 26.13	0.826	105.45 ± 30.86	0.93
Median MNCV	53.7 ± 3.1	<0.001	51.2 ± 5.7	0.033	46.5 ± 3.4	0.005
Median MNCA	8.9 ± 2.3	0.014	7.8 ± 2.8	0.398	6.9 ± 2.4	0.516
Median SNCV	51.8 ± 8.6	0.001	48.7 ± 8.8	0.086	41.9 ± 8.3	0.028
Median SNCA	21.4 ± 6.8	<0.001	17.0 ± 8.2	0.03	9.6 ± 5.4	<0.001
Tibial MNCV	44.5 ± 3.2	0.012	40.8 ± 5.89	0.007	35.8 ± 6.0	0.15
Tibial MNCA	8.7 ± 3.1	<0.001	8.2 ± 3.7	0.649	5.2 ± 3.5	0.02
Sural SNCV	50.3 ± 8.5	<0.001	47.5 ± 6.3	0.114	36.3 ± 3.9	0.002
Sural SNCA	13.2 ± 5.6	<0.001	10.4 ± 3.2	0.114	6.1 ± 3.1	<0.001
Insulin	16 (12.5%)	0.347	7 (20%)	0.576	13 (22.4%)	0.747
Sulfonylureas	32 (25.0%)	0.415	10 (28.6%)	0.589	21 (36.8%)	0.467
Metformin	46 (35.9%)	0.423	14 (40%)	0.71	25 (43.8%)	0.745
ACEI or ARB	33 (25.8%)	0.768	12 (34.3%)	0.896	19 (33.3%)	0.824

Results expressed as mean (standard deviation) or percentage

*DPN* diabetic peripheral neuropathy, *BMI* body mass index, *DBP* diastolic blood pressure, *SBP* systolic blood pressure, *FPG* fasting plasma glucose, *2* *h-PG* 2 h-postprandial glucose, *TC* total cholesterol, *TG* triglyceride, *HDL-C* high-density lipoprotein cholesterol, *LDL-C* low-density lipoprotein cholesterol, *HbA1c* glycosylated hemoglobin A1c, *GA* glycated albumin, *Cr* serum creatinine, *eGFR* estimated glomerular filtration rate, *MNCV* motor nerve conduction velocity, *MNCA* motor nerve conduction amplitude, *SNCV* sensory nerve conduction velocity, *SNCA* sensory nerve conduction amplitude, *ACEI* angiotensin-converting enzyme inhibitors, *ARB*, angiotensin receptor blocker, *P*_*1V3*_ non-DPN versus confirmed DPN, *P*_*1V2*_ non-DPN versus clinical DPN, *P*_*2V3*_ clinical DPN versus confirmed DPN

**Fig. 1:**
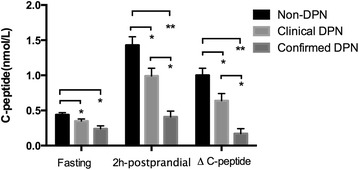
Fasting C-peptide, 2-hpostprandial C-peptideand ΔC-peptide concentration in non-DPN group, clinical DPN group and confirmed DPN group. ΔC-peptide, 2 h-postprandial C-peptide minus fasting C-peptide

Fasting C-peptide, 2-hpostprandial C-peptide and ΔC-peptide concentrations in the non-DPN group (0.44 ± 0.15, 1.42 ± 0.92 and 1.00 ± 0.81 nmol/L, respectively) were significantly higher than in the clinical DPN group (0.35 ± 0.17 nmol/L, *P* = 0.030; 0.99 ± 0.75 nmol/L, *P* = 0.024 and 0.64 ± 0.68 nmol/L, *P* = 0.040, respectively) and the confirmed DPN group (0.24 ± 0.14 nmol/L, *P* = 0.002; 0.41 ± 0.27 nmol/L, *P* < 0.001 and 0.17 ± 0.22 nmol/L, *P* < 0.001, respectively). Additionally, both the 2-h postprandial C-peptide and ΔC-peptide concentrations were higher in the clinical DPN group than in the confirmed DPN group (both *P* < 0.001; Fig. 1).

Fasting C-peptide, 2-h postprandial C-peptide and ΔC-peptide levels were all significantly correlated with diabetes duration (r_1_, −0.325; r_2_, −0.290 and r_3_, −0.234, respectively), FPG (r_1_, −0.271; r_2_, −0.572; and r_3_, −0.602, respectively), HbA1c (r_1_, −0.335; r_2_, −0.439 and r_3_, −0.419 respectively), GA (r_1_, −0.460; r_2_, −0.605 and r_3_, −0.522, respectively), median motor NCV (r_1_, 265; r_2_, 0.326 and r_3_, 0.297, respectively), ulnar motor NCV (r_1_, 286; r_2_, 0.401 and r_3_, 0.550, respectively) and sural sensory NCV (r_1_, 277; r_2_, 0.248 and r_3_, 0.226, respectively).

Multiple logistic regression analyses were performed to evaluate the risk factors associated with DPN (including both clinical and confirmed DPN) (Table 2). After adjusting for age, sex, diabetes duration, smoking status, BMI, SBP, ACEI/ARB use, FPG, HbA1c, TG and eGFR (model 2), the association between serum C-peptide levels and DPN remained statistically significant (odds ratio [OR], 0.329; 95% confidence interval [CI]), 0.107–0.903 for fasting C-peptide, *P* = 0.028; OR, 0.712; 95% CI 0.556–0.913 for 2-hpostprandial C-peptide, *P* = 0.007 and OR, 0.717; 95% CI 0.548–0.939 for ΔC-peptide, *P* = 0.016].

**Table 2:** Multiple regression analyses with the dependent variable of DPN and independent variable of C-peptide

	Model^1^		Model^2^	
	Odds ratio (95% confidence interval)	*P*	Odds ratio (95% confidence interval)	*P*
Fasting C-peptide	0.457 (0.234–0.892)	0.022	0.392 (0.170–0.903)	0.028
2 h-postprandial C-peptide	0.749 (0.621–0.904)	0.003	0.712 (0.556–0.913)	0.007
ΔC-peptide	0.733 (0.592–0.908)	0.004	0.717 (0.548–0.939)	0.016

*Model* ^*1*^ adjusted for age, sex and diabetes duration

*Model* ^*2*^ adjusted for age, sex and diabetes duration, smoking, systolic blood pressure, body mass index, angiotensin-converting enzyme inhibitors/angiotensin receptor blocker, fasting plasma glucose, glycosylated hemoglobin A1c, triglyceride, estimated glomerular filtration rate

Compared with the ΔC-peptide concentrations in quartile 1 (reference), patients in quartile 3 (OR 0.110; 95% CI 0.026–0.466; *P* = 0.003) and quartile 4 (OR 0.012; 95% CI 0.026–0.559; *P* = 0.007) had a lower risk of DPN (including both clinical and confirmed DPN) after adjusting for age, sex, diabetes duration, smoking status, BMI, SBP, ACEI/ARB use, FPG, HbA1c, TG and eGFR. Trend test analysis showed a statistical difference in the prevalence of DPN among quartiles 2, 3 and 4, compared to quartile 1 (*P* < 0.001; Table 3). However, the same analysis of fasting C-peptide and 2-h postprandial C-peptide did not show a significant difference among the quartiles.

**Table 3:** DPN risk in different ΔC-peptide quartiles

	Q1	Q2	Q3	Q4	*P* trend
	(≤0.22 nmol/L)	(0.23–0.59 nmol/L)	(0.60–1.15 nmol/L)	(>1.16 nmol/L)	
Odds ratio (95% confidence interval)	−	0.645 (0.191–2.175)	0.110 (0.026–0.466)	0.012 (0.026–0.559)	<0.001
*P*	−	0.479	0.003	0.007	

Multiple regression analyses and trend test analysis were used. The regression was adjusted for age, sex and diabetes duration, smoking, systolic blood pressure, body mass index, angiotensin-converting enzyme inhibitors/angiotensin receptor blocker, fasting plasma glucose, glycosylated hemoglobin A1c, triglyceride, estimated glomerular filtration rate. ΔC-peptide, 2-hpostprandial C-peptide minus fasting C-peptide

## Discussion

This study suggested a close relationship between the serum C-peptide concentrations and DPN in community-based Chinese type 2 diabetes patients. The decrease in ΔC-peptide was strongly associated with the prevalence of DPN after adjusting for other variables.

Some studies showed that serum C-peptide concentration had a protective effect on neuropathy. In a large clinic-based cohort of 471 type 1 diabetic patients, higher values conferred a protective effect (OR 0.59; 95% CI 0.37–0.94) on diabetes microvascular complications including autonomic neuropathy, compared to C-peptide values in the lowest tertile (<0.06 nmol/L) [7]. However, the role of C-peptide concentrations for DPN in type 2 patients was still controversial. A study in Korea showed that the risk for diabetic neuropathy was associated with the lower fasting serum C-peptide quartile and lower ΔC-peptide quartile in type 2 diabetic patients after adjusting for multiple confounding factors [11]. A retrospective cohort study with a median follow-up of 14 years showed that the risks for incident neuropathy were negatively associated with the highest C-peptide tertile (OR 0.39; 95% CI 0.25–0.61) [12]. In a Chinese study that included hospitalized patients with type 2 diabetes, Zhao et al. [13] concluded that a higher level of area under the curve of C-peptide [AUC (C-pep)] was inversely associated with the prevalence of neuropathy. However, Sari et al. [14] demonstrated that C-peptide did not correlate with sensorial neuropathy. Moreover, another study found that patients with parasympathetic neuropathy had elevated fasting plasma C-peptide (*P* < 0.001) [15]. Our study focused on the relationship between C-peptide concentration and DPN in community-based Chinese patients, and the results suggested that the fasting C-peptide, 2-h postprandial C-peptide and ΔC-peptide concentrations were negatively associated with DPN after adjusting for multiple confounders.

In our study, DPN was associated with poor glycemic control as reflected by HbA1c, old age and longer diabetes duration. With increased diabetes duration, the islet function diminishes gradually, resulting in reduced C-peptide and insulin levels and the prevalence of DPN increases. Therefore, we conducted a different analysis to eliminate the effect of age and disease duration on the results. For example, under the circumstances of no obvious difference of age and diabetes duration among the three groups, the C-peptide differed significantly. Furthermore, multiple logistic regression analysis showed a strong relationship between C-peptide and DPN even after adjustment for confounding factors including age, sex, diabetes duration, smoking status, BMI, SBP, ACEI/ARB use, FPG, HbA1c, TG and eGFR, indicating that C-peptide was independently associated with DPN. These results are consistent with other studies that included patients with lower HbA1c levels or shorter disease duration or younger age [10–12].

The beneficial effects of C-peptide on the prevention of diabetes complications in type 1 diabetes patients have been confirmed by various studies [8, 9]. In contrast, the role of C-peptide is not well-defined in type 2 diabetes. Experimental studies in type 1 diabetes showed that C-peptide specifically bound to cell surfaces, acting via a G-protein-related receptor; it also led to autophosphorylation of the insulin receptor in the presence of insulin [20]. Moreover, C-peptide stimulated p38 MAP-kinase and PI-3 kinase activities, and diminished the activation of JNK phosphorylation with subsequent effects on Na^+^/K^+^-ATPase activity and nitric oxide (NO) [21, 22]. C-peptide also ameliorated the altered expression of insulin-like growth factor-1, nerve growth factor and neurotrophin-3 and their respective receptors, which corrected neurofilament (NF) and tubulin mRNA, and protein expression, as well as normalized the aberrant phosphorylation of NFs [23]. C-peptide also stabilized the attachment of the α-Na^+^-channels at the nodal axolemma, and furthermore, it prevented a breach of the paranodal ion-channel barrier. These results correlate with the corrected effects of C-peptide on nodal and paranodal structural integrity [24, 25]. In addition to the direct effect of the C-peptide on DPN, the residual beta cell function as represented by C-peptide concentrations also plays an important role. This highlighted the importance of some treatment strategies, such as avoiding drugs that overstimulate beta cells and initiating insulin therapy at an appropriate time to preserve endogenous beta cell activity in the progression of type 2 diabetes.

This study has some limitations that should be taken into account. First, due to its cross-sectional nature, we could not determine the causal relationship between the serum C-peptide levels and DPN. Thus, prospective studies are needed to confirm the protective effects of C-peptide on DPN. Second, the relationship between C-peptide levels and severity of DPN was not analyzed. Third, the clinical confirmed DPN should be validated by small fibre examinations, such as corneal confocal microscopy. Finally, the relationship between C-peptide levels and other microvascular complications was not investigated. This relationship will be evaluated in further studies.

## Conclusions

Serum C-peptide levels were significantly associated with DPN in community-based Chinese type 2 diabetic patients. In patients with low C-peptide levels, a high prevalence of DPN was observed. Decreased ΔC-peptide levels were strongly associated with the prevalence of DPN after adjusting for other confounding variables. Thus, C-peptide might play a potential protective effect on the occurrence of DPN in patients with type 2 diabetes. Large prospective studies should be conducted to define the causal correlation between C-peptide and DPN in type 2 diabetes mellitus.

## Abbreviations

DPN: diabetic peripheral neuropathy
HbA1c: hemoglobin A1c
eGFR: estimated glomerular filtration rate
BMI: body mass index
SBP: systolic blood pressure
DBP: diastolic blood pressure
GA: glycated albumin
FPG: fasting plasma glucose
2 h-PG: 2 h-postprandial plasma glucose
Cr: creatinine
TC: total cholesterol
TG: triglyceride
HDL-C: high-density lipoprotein–cholesterol
LDL-C: low-density lipoprotein–cholesterol
GFR: glomerular filtration rate
MDRD: modification of diet in renal disease
NSS: neuropathy symptom score
NDS: neuropathy disability scores
NCV: nerve conduction velocity
MNCV: motor nerve conduction velocity
MNCA: motor nerve conduction amplitude
SNCV: sensory nerve conduction velocity
SNCA: sensory nerve conduction amplitude
OR: odds ratio
CI: confidence interval
NO: nitric oxide
NF: neurofilament
